# Purification and Activity of the Second Recombinant Enzyme for Biodegrading Linearized Microcystins by *Sphingopyxis* sp. USTB-05

**DOI:** 10.3390/toxins15080494

**Published:** 2023-08-04

**Authors:** Junhui Teng, Meijie Song, Qianqian Xu, Qianwen Zou, Haiyang Zhang, Chunhua Yin, Xiaolu Liu, Yang Liu, Hai Yan

**Affiliations:** School of Chemistry and Biological Engineering, University of Science and Technology Beijing, Beijing 100083, China; m202120914@xs.ustb.edu.cn (J.T.);

**Keywords:** microcystins, biodegradation, linearized microcystinase, purification, activity

## Abstract

Hepatotoxic microcystins (MCs) are produced and released by the harmful bloom-forming cyanobacteria, which severely threaten drinking water safety and human health due to their high toxicity, widespread distribution, and structural stability. The linearized microcystinase (MlrB) further hydrolyses the poisonous linearized MCs produced by the microcystinase-catalysed MCs to form tetrapeptides. Here, the purification and activity of MlrB were investigated. The results showed that the linearized products generated by 12.5 mg/L MC-LR and MC-RR were removed by purified recombinant MlrB at a protein concentration of 1 mg/L within 30 min. The high catalytic activity of MlrB can be obtained via heterologous expression and affinity purification, which lays the foundation for further studies on the properties and mechanism of MCs biodegradation enzymes.

## 1. Introduction

With the intensification of global warming and water eutrophication, the pollution by cyanobacteria bloom is increasing and becoming more serious all over the world [1,2,3]. Aggravated blooms of cyanobacteria reduce dissolved oxygen in the water, deteriorate water quality, and seriously disrupt the balance of aquatic ecosystems. In particular, cyanobacteria produce and release a wide range of algal toxins that not only poison aquatic organisms but also pose a further threat to human health through drinking water [4]. Currently, the harm of cyanobacteria blooms to water resources, human health, and aquatic flora and fauna is still a vital matter of global concern.

Cyanobacterial toxins are kinds of toxic metabolites released by the cyanobacteria’s cell death or lysis. The typical algal toxins consist of microcystins (MCs), nodularins (NODs), cylindrospermopsins (CYN), anatoxin-α, homoanatoxin-α, and saxitoxins, among others [5]. MCs, NODs, and CYN are a set of major cyclic polypeptide hepatotoxins, of which MCs are considered to be the most widespread and severe toxic [1,6,7]. MCs are generally formed by the species of genera *Microcystis*, *Planktothrix*, and *Anabaena*. Moreover, *Nostoc*, *Oscillatoria*, *Phormidium*, *Pseudanabaena*, *Hapalosiphon*, *Fischerella*, and *Synechocystis* can also produce them [8].

MCs are types of monocyclic heptapeptide hepatotoxins (-D-Ala-L-X-D-MeAsp-L-Z-Adda-D-Glu-Mdha) with molecular weights between 900 and 1100 Dalton (Da), containing three specific amino acids: D-erythro-β-methylaspartic acid (MeAsp) at position 3, 3-amino-9-methoxy-2,6,8-trimethyl-10-phenyl-4,6-decadienoic acid (Adda) at position 5, and N-dehydroalanine (Mdha) at position 7 [9]. There are two variable amino acids at positions 2 and 4 in the chemical molecule structure of MCs. To date, more than 270 different isomers of MCs have been identified, based on the degree of methylation, hydroxylation, epimerization, and variable amino acids [10]. Except that, MC-LR with the variable amino acids, leucine (L) and arginine (R), MC-RR with two variable amino acids arginine and MC-YR with tyrosine (Y) and arginine (R) are the more common and highly toxic toxins than others [11]. Free Adda is not toxic in itself, but its stereospecific structure with other amino acids results in toxic activity with the liver being the main target organ [12,13]. What is more, MCs inhibit serine/threonine protein phosphatases PP1 and PP2A, which not only affects the regulation of cellular protein phosphorylation and promotes apoptosis [8] but also contributes to the morphological transformation of microtubules, causing cell destruction and cytoskeletal damage [14]. The prolonged and frequent exposure to low concentrations of MCs can eventually lead to uncontrolled cell proliferation in the human body and promote the occurrence and development of tumors, leading to primary liver cancer [15,16].

At present, the natural degradation of MCs in aquatic environments is very slow due to the structural stability of inherited ring structure and spaced double bonds, and the physical methods and chemical reactions cannot provide a cheap, easy, and uncontaminated way of removing MCs [17,18,19]. Compared with other methods, microbiological methods have the advantages of low cost, high efficiency, safety, non-pollution, and eco-remediation, and are considered to be the most promising method for removing MCs [20,21,22]. The first *Sphingomonas* sp. (ACM-3962) with the ability to degrade MC-LR was isolated from natural waters by the Australian scientist Jones and his colleagues in 1994 [23]. Bourne et al. investigated the pathway and molecular mechanism of enzymatic biodegradation of MC-LR by *Sphingomonas* sp. ACM-3962 and found that at least four enzymes, MlrA, MlrB, MlrC, and MlrD, were involved in the biodegradation of MC-LR [24,25]. Furthermore, dipeptidases (MlrE) and D-aminoacylases (MlrF), which encoded genes located near the *mlr* gene cluster, are thought to be associated with hepatotoxin biodegradation [26]. MlrA can linearize the cyclic structure of MCs and reduce their toxicity, and the linearized MCs were still found to have certain toxicity [24]. Removal of linearized MCs is urgent. As far as we know, the MlrB and tetrapeptidase (MlrC) can further degrade linearized MCs, which has been reported [20,27,28,29].

Dziga et al. first purified heterologously expressed MlrA by C-terminal histidine (His) tagging and found that MlrA activity measured in *Escherichia coli* (*E. coli*) BL21 (DE3) extracts was 6800-fold higher than wild *Sphingomonas* strains, and transformed *E*. *coli* cells were nearly 250-fold more efficient at MC linearization than unmodified cells of *Sphingomonas* sp. [27]. This suggests that this may be a key advantage of using modified microorganisms for bioremediation applications and also opens up new avenues for the study of enzyme molecules. To date, the purification and mechanism of the activity of linearized microcystinase have only been reported in two papers. Dziga et al. validated the key sites, despite the fact that the purified enzyme solution was mixed with a large number of other proteins, mainly by mutating serine at position 77 and lysine at position 80 to alanine [20]. Wei et al. used a combination of in vitro experiments and computer simulations to characterize and discuss the molecular mechanism of linearized MC-LR degradation by linearized microcystinase [30]. Meanwhile, our research group has successfully screened several strains for MCs biodegradation, such as *Sphingopyxis* sp. USTB-05 in 2002 [31]. Three MCs degradation genes were cloned, and recombinant enzymes with continuous catalytic degradation activity of MCs were expressed [32,33,34,35,36]. We successfully purified MlrA and MlrC and simulated the 3D structures to study the active sites of both enzymes [36,37]. However, there are still many gaps in the further degradation of the MCs by the purified MlrB. On the basis of previous research, we found that crude recombinant enzyme MlrB always mixed with complex cellular contents of host *E. coli*, which influence the accurate quantification and in-depth study of MlrB [30,35]. In this study, the effective recombinant MlrB from *Sphingopyxis* sp. USTB-05 has been purified successfully and shown a high level of biodegradation activity, which lays a significant foundation for further study on the mechanism of enzymatic biodegradation.

## 2. Results

### 2.1. Purification of MlrB

**Figure 1:**
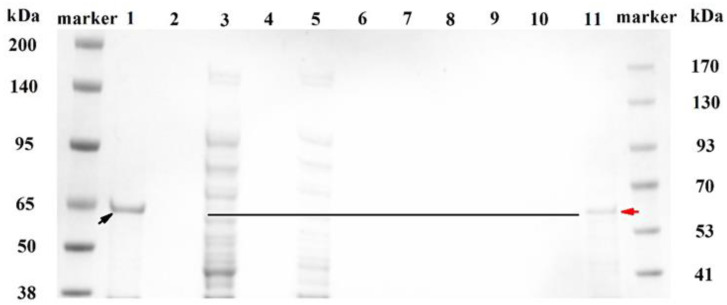
SDS-polyacrylamide gel electrophoresis (SDS-PAGE) analysis for each fraction during the purification. Lane marker: Molecular weight standards; Lane 1: Precipitation fraction of recombinant cell extract; Lane 3: Supernatant fraction of recombinant cell extract; Lane 5: Flow-through fraction after His-labeled MlrB binding on the resin; Lane 7 and 8: Flow-through fraction after washing the resin; Lane 9: Fluted His-labeled MlrB for the first time; Lane 10: Eluted His-labeled MlrB for the second time; Lane 11: Purified His-labeled MlrB protein after ultrafiltration. The inclusion bodies and enzyme bands of MlrB are marked with black and red arrows, respectively. The black line indicates the location of the enzyme.

The purification of MlrB was performed as follows: Affinity chromatography of a nickel column with His-tagged MlrB, gradient elution of impurity proteins, collection of target proteins, and removal of imidazole by ultrafiltration. Figure 1 shows the different fractions during the purification via SDS-polyacrylamide gel electrophoresis (SDS-Page) analysis. Although the inclusion body of MlrB in precipitation marked with a black arrow in Lane 1 was the major pattern, soluble MlrB in supermatant was sufficient to be used (Lane 3) for further purification. Separation of His-labeled MlrB was separated by Ni-chelating affinity chromatography. In the next step, the non-specific adsorbed proteins were washed with eluates containing different concentrations of imidazole. For the first time, the non-specific adsorbed proteins were washed with 35 mM imidazole and then again with 60 mM imidazole. The wash solution was collected separately and analyzed by Nanodrop until the value of optical density 280 (OD_280_) reached baseline. The results indicated that the non-specific adsorbed proteins had been completely removed, consistent with the SDS-PAGE results where neither the non-specific adsorbed proteins nor the target proteins were visible (Lanes 7–8). Then, the target protein was eluted from the resin column with an eluent containing 125 mM imidazole. This step was repeated twice to obtain sufficient target protein. However, the eluted His-labeled MlrB bands were still not visible due to the low concentration of the protein (Lanes 9–10). Ultrafiltration enrichment of His-labeled MlrB made the MlrB band clearly visible (Lane 11). Meanwhile, the imidazole, which affected the stability and activity of MlrB, was removed during the ultrafiltration. The expected band of MlrB was marked with a red arrow. It was found at approximately 65 kilo Dalton (kDa), which is consistent with a theoretical 60 kDa. The purification of the MlrB enzyme was successful, and the enzyme could be available for further studies on pure enzyme activity.

### 2.2. Enzymatic Activity of Purified MlrB

**Figure 2:**
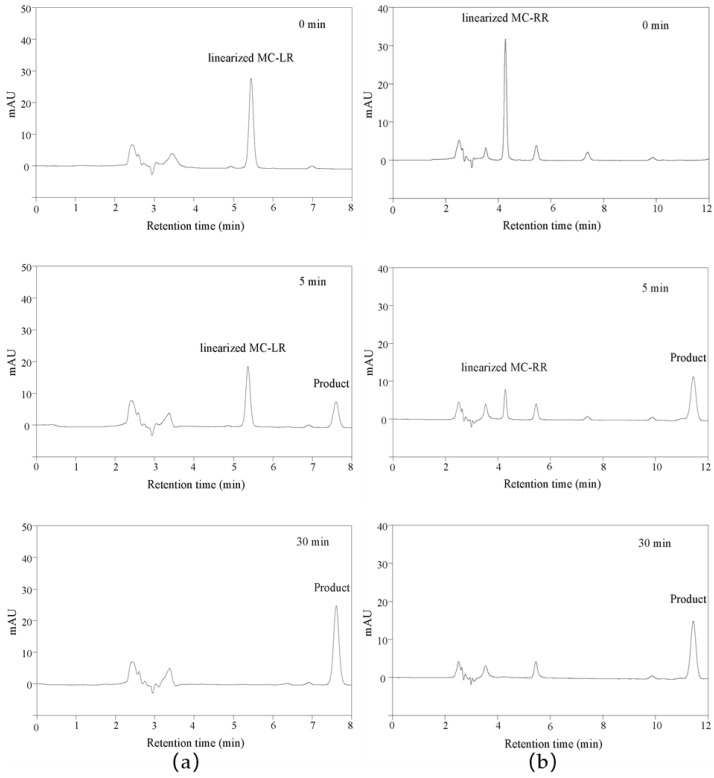
High-performance liquid chromatography profiles for linearized MC-LR and MC-RR biodegradation by cleaned linearized microcystinase MlrB. A coordinate system was established based on millimolar absorption units (MAU) as the vertical coordinate and retention time as the horizontal coordinate. (**a**) Demonstration of the biodegradation of linearized MC-LR by MlrB to produce tetrapeptides at three stages, 0, 5, and 30 min, respectively. (**b**) Demonstration of the biodegradation of linearized MC-RR by MlrB to produce tetrapeptides at three stages, 0, 5, and 30 min respectively.

In previous articles, the mass spectrometry of linearized MC-LR and MC-RR biodegradation products was discussed in detail to verify the function of MlrB, which is responsible for the degradation of MCs into tetrapeptides [33,38]. For this reason, only HPLC was used to monitor the entire catalytic degradation process. Apparently, the retention time of the linearized MC-LR was about 5.4 min, as shown in Figure 2a. The peak of linearized MC-LR decreased as the reaction time extended. At the same time, the peak of tetrapeptide appeared at 7.6 min and gradually increased with the increasing reaction time. In Figure 2b, within 30 min of the reaction time, the linearized MC-RR with a retention time of 4.2 min continued to decrease as the biodegradation was processed, while the tetrapeptide with a retention time of 11.4 min appeared accordingly and continued to increase.

**Figure 3:**
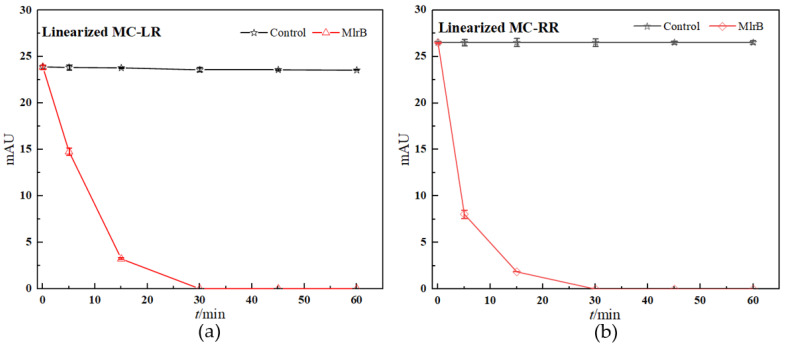
Linearized MCs biodegradation kinetics by purified MlrB: (**a**) For linearized MC-LR, (**b**) for linearized MC-RR. In the control group, PBS was filled into the reaction system instead of MlrB. The nodes in the control group are marked with black squares. The nodes in the biodegradable group are marked with red dots. Each node was run in parallel three times, and the average of these was taken as the final data. Note: The error bars represent standard deviation (SD)+ standard error of the mean (SE) of three replicates in each group.

**Figure 4:**
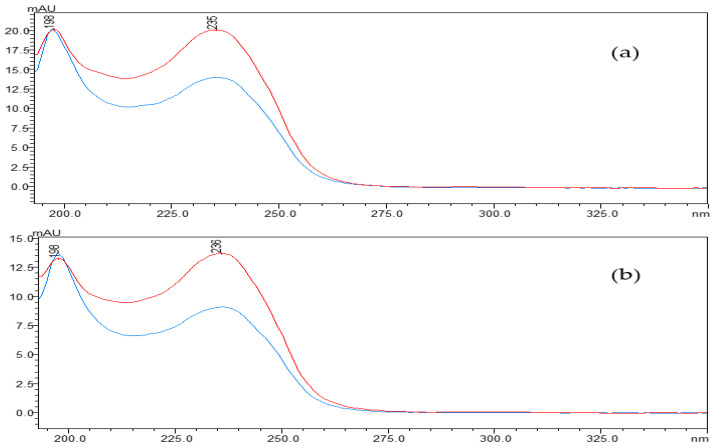
The UV spectra profiles of linearized MCs: (**a**) For linearized MC-LR, (**b**) for linearized MC-RR. linearized MC-LR or MC-RR (blue) and its product (red). The spectra were taken at intervals of 5 nm.

The concentration of linearized MCs fell off a cliff within the first five minutes of the reaction, in which the linearized MC-LR and MC-RR were rapidly broken down by the MlrB into tetrapeptides, respectively. The biodegradation curve flattened over the next 10 min. By this time, most of the toxin had been broken down by the enzymes. After 30 min, the biodegradation curve reached and maintained its lowest point, indicating that the entire biodegradation process was complete. The results showed that the linearized toxins of 12.5 mg/L MC-LR and MC-RR were removed by 1 mg/L purified enzyme MlrB in only 30 min. (Figure 3). Finally, pure enzyme MlrB with high biodegradation activity through several series of purification steps was obtained. The linearized MCs and the tetrapeptide still contained the Adda structure, so the UV scan spectrum of the tetrapeptide peak in the wavelength range of 200 to 350 nm showed a similar UV spectrum to the linearized MC-LR and MC-RR, respectively (Figure 4).

## 3. Discussion

Intermediates are difficult to be tracked by using naturally sourced strains in the MC biodegradation because that is a rapid and continuous process accompanying production and biodegradation of the intermediates [20]. Several enzymes are usually involved in the process. Fortunately, target enzymes can generally be produced in heterologous systems. Wang et al. found that the heterologously expressed recombinant enzyme had a higher degradative capacity by comparing the activity of crude enzyme extract and intact recombinant enzyme against MC-RR [39]. This conclusion is strongly supported by the biodegradation experiments of MC-LR carried out by Dziga et al. using heterologous MlrA extracts in comparison with the use of natural strains or the immobilized expression of whole-cell MlrA in *E*. *coli* [40]. Based on a genetic engineering approach, in short, the gene sequence of the target enzyme was inserted into an expression vector using recombinant DNA technology, and then the constructed recombinant plasmid was transferred into the host organism to produce the enzyme [41]. Therefore, intermediates can be easily obtained by using individual enzymes to biodegrade MCs step by step. The characteristics of the enzymes, for instance, structure, activity ability, and biodegradation mechanism, can also be further studied [20].

Immobilized metal affinity chromatography (IMAC) can be used to purify proteins with surface-exposed mono- or oligo-histidine residues [42,43]. Bourne et al. speculated that a signal peptide consisting of 26 amino acid residues at the carboxyl terminus of MlrA would be cut off as the protein matures, resulting in the absence of labeling [25]. For this reason, the pET-30a vector, which contains a histidine tag at each end, was chosen to construct the recombinant plasmid pET-30a/*mlrB*. His-labeled MlrB was obtained by expression in *E. coli* BL21 (DE3), which had specific adsorption with Ni^2+^ in the affinity chromatography column and could be efficiently eluted by adding free imidazole or reducing the pH of the column buffer [44]. It has been shown that a gradient elution method can be used to obtain repeatable target proteins with a purity of over 90% [45]. The non-specific adsorption was reduced effectively during this process. In order to obtain high purified MlrB, we set up eight gradients with different imidazole concentrations ranging from 20 to 100 mM. Ultrafiltration was used to remove high concentrations of imidazole to avoid interfering with enzyme activity and concentrate the protein. According to the SDS-PAGE results, the targeted bands were invisible in Lanes 9 and 10 due to the low concentration, but Lane 11 was clearly visible after ultrafiltration. Finally, we harvested purified MlrB with a high level of activity, which was also confirmed by activity experiments.

The *mlr*-dependent biodegradation pathway, in which related enzymes were encoded by the *mlrABCD* gene cluster, is the main and best-known biological method for hepatotoxin biodegradation [46]. Bourne et al. tested the 50% inhibitory concentrations (IC50) of MC-LR and its two degradation products for crude chicken brain protein phosphatase. The results showed that the IC50 was 0.6 nM for cyclic MC-LR, confirming that the toxin poses a considerable risk to human health [24]. Subsequently, following the action of the first biodegradable enzyme, the cyclic MC-LR was cut and opened at the Adda-Arg link, resulting in a linearized MC-LR product with the IC50 of 95 nM and an approximately 2100-fold reduction in toxicity [24,27,29,41]. Then, the MlrB enzyme hydrolyzed the peptide bonds of the linearized MC-LR to produce a product called tetrapeptide (Adda-D-Glu-Mdha-D-Ala-OH). Tetrapeptide is also toxic. However, in any case, the biodegradation of linearized MC-LR with MlrB is an essential part of the process. Finally, MlrC hydrolyzes the tetrapeptide to produce the non-toxic Adda, which eventually enters the phenylacetic acid pathway for complete degradation. MlrB enzyme was considered to belong to the serine hydrolase family and had a high sequence similarity to members of the penicillin recognition enzyme family with the conserved sequence Ser-Xaa-Xaa-Lys [25,28,47]. Information on the similarities and differences between the different strains of MlrB enzymes was obtained from the AlphaFold Protein Structure Database. The MlrB enzyme and the gene coding for it were the main search terms and were further selected on the basis of their sequence lengths and the per-residue confidence score (pLDDT) indices of their 3D structures. Among them, six strains possessing the same amino acid sequence length are *Sphingomonas* sp. ACM-3962, *Novosphingobium* sp. MD-1, *Novosphingobium* sp. THN1, *Sphingopyxis* sp. X20, *Novosphingobium* sp. ERW19, and *Sphingopyxis* sp. C-1. The degradation gene *mlrB* derived from *Sphingopyxis* sp. USTB-0*5* contains 1623 base pairs and encodes a serine carboxypeptidase containing 541 amino acid residues. In the degradation reactions with linearized MC-LR or MC-RR, MlrB cleaves the peptide bond between alanine (Ala) and leucine (Leu), degraded to tetrapeptide. Our results were consistent with the previous studies [20,24,29], which also reported that linearized MC-LR was biodegraded to tetrapeptide by some bacteria according to high-performance liquid chromatography-tandem mass spectrometry (HPLC/MS) data. As shown in Figure 4, the scanning spectrograms also reflect the same phenomenon. The maximum absorption peaks of both linearized MCs and tetrapeptides were found to be around 238 nm. The similarity between the two scans was very high (Figure 3 and Figure 4), indicating that the linearized MCs and tetrapeptide have similar molecular structures. All of the results showed that the purified enzyme MlrB can catalyze the degradation of linearized MC-LR and MC-RR efficiently.

In conclusion, we expressed and purified the linearized microcystinase MlrB derived from *Sphingopyxis* sp. USTB-05 successfully. The purified recombinant MlrB had high activity for biodegrading linearized microcystin MC-LR and MC-RR. There are more promising applications in research and industry: (1) Using pure MlrB to study the biodegradation mechanism of MCs, (2) bringing industrially produced enzymes into direct contact with polluted water to exploit their biodegrading ability, (3) using enzyme immobilizing techniques to immobilize MlrB on suitable media to improve its recycling rate, etc. This study provides a good foundation for further research and application.

## 4. Materials and Methods

### 4.1. Strains and Reagents

The strain used was *Sphingopyxis* sp. USTB-05, capable of biodegrading MCs and NODs, previously isolated from Dianchi, China [48]. To construct a recombinant protein-expressing strain carrying the mlrB gene, we used the plasmid-amplified vector strain E. coli TOP10 and the expression strain *E. coli* BL21, which were purchased from Sangon Biotechnology Ltd. in Shanghai, China. Naturally, the cyclic vectors pET-30a having two histidine tags, both BamH I and Xho I restriction enzymes, the plasmid isolation kit, the polymerase chain reaction (PCR) kit, and the Ni-NTA protein purification kit were also all obtained from Sangon in Shanghai, China. The recombinant strains were grown in Luria-Bertani (LB) medium at 200 r/min at 37 °C. Ampicillin and Kanamycin were purchased from Biorigin (Beijing) Inc. (Beijing, China), which can select bacteria and prevent contamination during growth. Standard MC-LR (C_49_H_74_N_10_O_12_) of 99% purity was purchased from Shanghai Aibixin Biotechnology Co. Imidazole and standard MC-RR (C_49_H_75_N_13_O_12_, 95% purity) were purchased from Shanghai Malin Biochemistry Co., Shanghai, China. All other reagents used in the purification process were analytic grade.

### 4.2. Cloning and Expression of the Linearized-Microcystinase Gene mlrB

The linearized-microcystinase gene *mlrB* was amplified from *Sphingopyxis* sp. USTB-05 by using PCR. The primers were 5′-GGATCCATGACTGCAACAAAGCTTTTCCTGG-3′ (forward) and 5′-CTCGAGCTACGGAAGCCGTCTGAACTCTAT-3′ (backward). The forward and reverse primers both contained restriction sites (underlined) of BamH I and Xho I, respectively. The same methods as described in the article were used for the PCR reaction and plasmid transformation [33]. In the first place, the pET-30a vector was linearized by double digestion with BamH I and Xho I. Then *mlrB* gene was connected to the pET-30a vector. The constructed plasmids were inserted into the cloned host *E. coli* TOP10 and positive clones were selected, and gene sequencing was performed by Shanghai Sangon Biotechnology Co., Ltd. in China to confirm the success of the transformation. The expression plasmid constructs extracted from *E. coli* TOP10 were transformed into *E. coli* BL21 (DE3) and exposed to antibiotic selection. Positive clones were also screened out and confirmed by sequencing.

MlrB expression follows the previously described method [36]. The recombinant *E. coli* BL21 (DE3)/pET-30a/*mlrB*/was inoculated in a 10 mL culture tube supplied with 3 mL LB medium (0.5% yeast extract, 1% peptone, and NaCl, pH 7.0) containing 50 μg/mL kanamycin and cultivated overnight at 37 °C with a rotational speed of 200 rpm. Overnight culture was transferred to 100 mL LB medium containing 50 μg/mL kanamycin with 1% (*v*/*v*) inoculum and incubated at 37 °C for 3 h with a rotational speed of 200 rpm. Protein expression was induced by adding 0.2 mM isopropyl-β-d-thiogalactopyranoside (IPTG) to the culture while the bacterial density measured at 600 nm reached 0.6. After incubation, the cells were harvested by centrifugation (10 min, 14,000rpm, 4 °C) and then washed with phosphate-buffered solution (PBS, pH 7.4) several times and frozen. All the following steps were performed on ice and with cooled (4 °C) buffers. Subsequently, the cell pellet was resuspended in PBS pH 7.4. The cells were disrupted using sonification, and cell debris was removed by centrifugation (20 min, 14,000 rpm, 4 °C). The filtrated supernatant was collected as a cell-free extract for further purification. The recombinant proteins were detected again by SDS-polyacrylamide gel electrophoresis (SDS-PAGE) on 10% polyacrylamide separation gel stained with Coomassie brilliant blue using whole-cell lysates and cell fractionation. Protein content was measured using the Bradford assay [49].

### 4.3. Purification of Linearized-Microcystinase MlrB

His-labeled MlrB was purified from the filtered supernatant by use of a Ni-NTA prepacked column. The conditions were as described in the instructions. The polyhistidine tags exhibit the closest interaction with immobilized metal ion matrices, as electron donor groups on the histidine imidazole ring preferentially form coordination bonds with immobilized transition metal ions. A 6% highly cross-linked agarose was used as a substrate, and a tetra-liganded nitrogen triacetic acid (NTA) chelating nickel ion was covalently coupled by chemical means [50,51,52]. In the first place, the column was pre-equilibrated with 10× column volume of PBS (pH 7.4) [50]. Then, the cell-free supernatants were transferred to the resin to allow maximum binding of the target protein to the nickel ion and then recovered in tubes for further analysis. The columns were cleaned with PBS (pH 8) containing 35 and 60 mM imidazole, respectively, until the absorbance of the flow-through fraction at 280 nm was close to the baseline. The bound MlrB was eluted from the resin with 10 column volumes of 125 mM imidazole eluent. This procedure was repeated twice. Separate tubes were used to collect each fraction. SDS-PAGE was applied to the flow-through fractions and extracted MlrB. A 10 kilodalton (kDa) centrifugal ultrafiltration tube was used to enrich MlrB by ultrafiltration. Finally, the imidazole was removed by ultrafiltration with PBS once again. As the His-tags were small and did not affect the active function of the enzyme, there was no need to excise them.

### 4.4. Assessing the Biodegradability of Pure Linearized-Microcystinase MlrB

The purified MlrB was checked for biodegradation activity on the basis of three replicate experiments in both control and treatment groups. The linearized MCs used in this study were prepared based on our previous method [35]. More exactly, 12.5 mg/L MC-LR and MC-RR produced corresponding linearized toxins in the presence of MlrA, respectively. In the treatment group, the initial linearized MC-LR or MC-RR was biodegraded by purified MlrB with protein concentration at 1 mg/L. In the control group, PBS was filled into the reaction system instead of MlrB. The reaction condition is 30 °C with a shaking rate of 200 rpm. To terminate the reaction, 1% concentrated hydrochloric acid (HCl) was poured into the sample. Impurities in the samples were removed by centrifugation at 14,000 rpm for 10 min, and the supernatant was stored at −20 °C until analysis. The treated samples were analyzed with high-performance liquid chromatography (Shimadzu LC-20AT, Shimadzu Co., Ltd., Kyoto, Japan) with an ultraviolet diode array detector at 238 nm and an Agilent TC–C18 column (4.6 × 250 mm) (Agilent, 1200 series, Wilmington, DE, USA). Linearized toxins were quantified accurately. The mobile phases for linearized MC-LR and MC-RR were 36% and 40% (*v*/*v*) acetonitrile water solution mixed with 0.05% (*v*/*v*) of trifluoroacetic acid, respectively. The maximum flow rate was 1.0 mL/min, and the injection volume was 60 µL, ensuring a sample volume of 20 µL in the injection loop.

## Data Availability

Not applicable.
